# Supplementary figures and images for: Comprehensive In Silico Analysis and Transcriptional Profiles Highlight the Importance of Mitochondrial Dicarboxylate Carriers (DICs) on Hypoxia Response in Both Arabidopsis thaliana and Eucalyptus grandis
Source: Plants (Basel). 2022 Jan 11;11(2):181. doi: 10.3390/plants11020181 (PMC8779624; doi:10.3390/plants11020181)

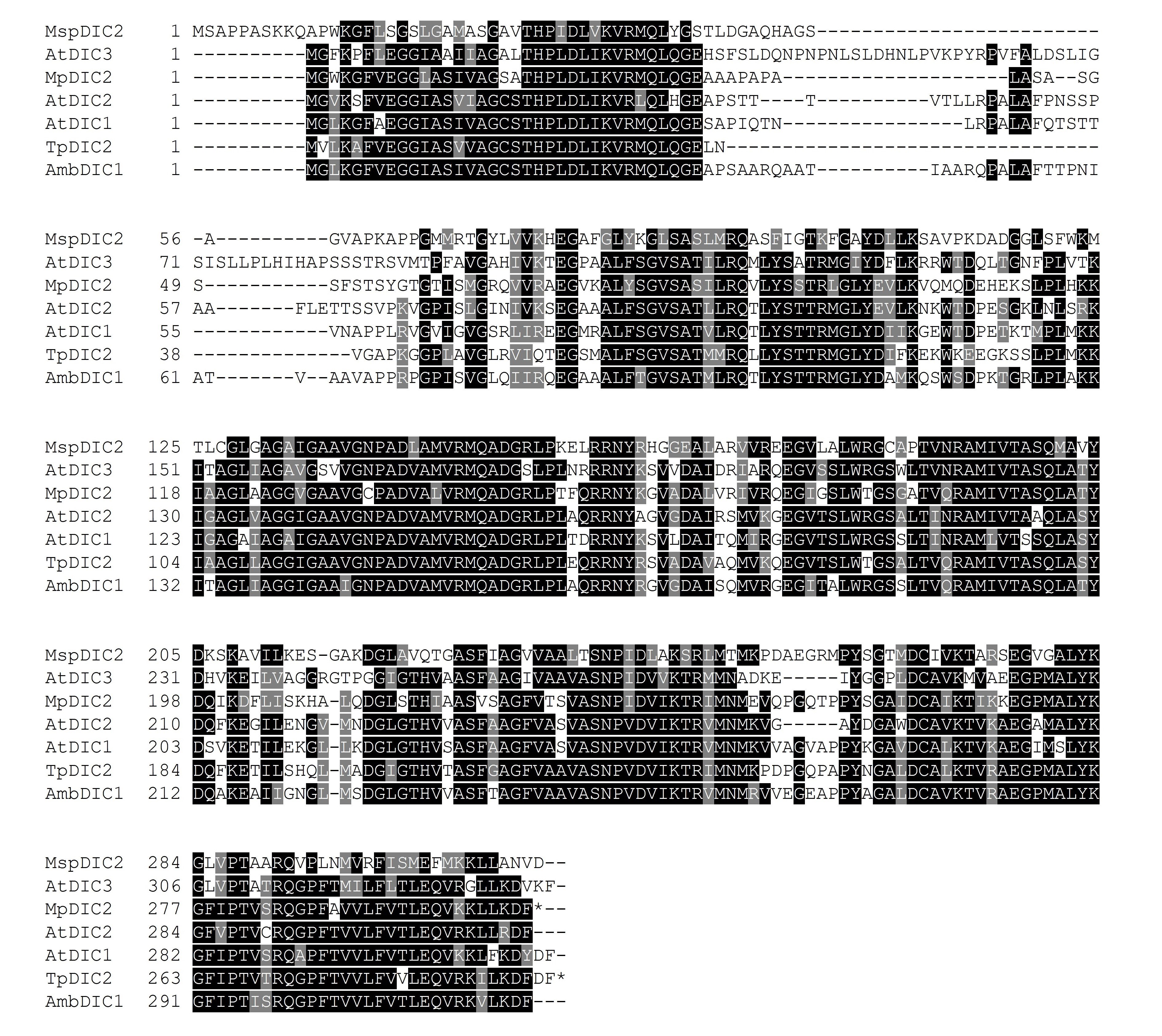

Supplement: Supplementary file 1 [file plants-11-00181-s001.zip › Figure S1.jpg]

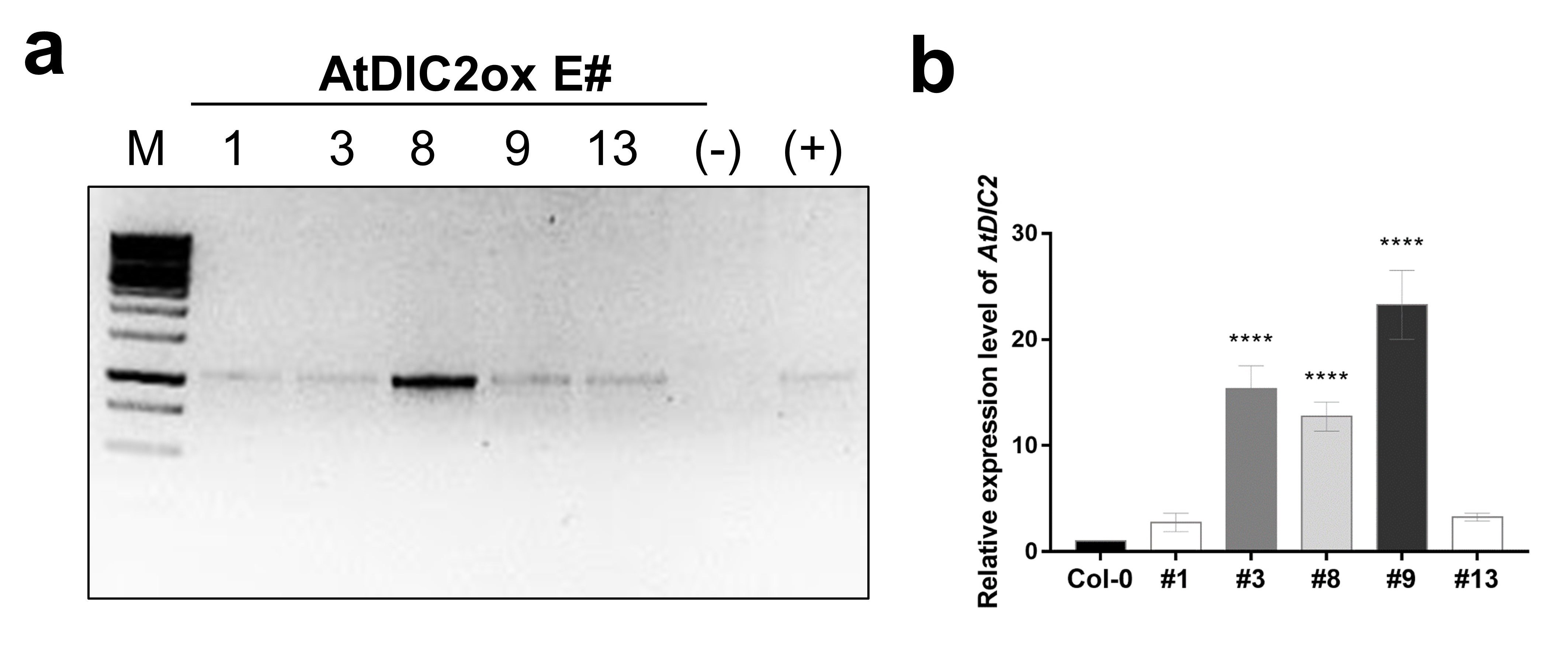

Supplement: Supplementary file 1 [file plants-11-00181-s001.zip › Figure S2.jpg]
